# Body Image and Nutritional Status Are Associated with Physical Activity in Men and Women: The ELSA-Brasil Study 

**DOI:** 10.3390/ijerph120606179

**Published:** 2015-05-29

**Authors:** Carolina G. Coelho, Luana Giatti, Maria D. C. B. Molina, Maria A. A. Nunes, Sandhi M. Barreto

**Affiliations:** 1 Public Health Postgraduate Program, Research Group on Epidemiology of Chronic and Occupational Diseases (GERMINAL), Universidade Federal de Minas Gerais, Belo Horizonte, 31270-901, Brazil; E-Mails: luana.giatti@gmail.com (L.G.); sandhi.barreto@gmail.com (S.M.B.); 2 Nutrition School, Universidade Federal de Ouro Preto, Ouro Preto, 35400-000, Brazil; 3 Postgraduate Program in Public Health, Universidade Federal do Espírito Santo, Vitória, 29043-900, Brazil; E-Mail: mdmolina@uol.com.br; 4 Postgraduate Program in Epidemiology, Universidade Federal do Rio Grande do Sul, Porto Alegre, 90035-003, Brazil; E-Mail: maanunes@gmail.com

**Keywords:** ELSA-Brasil, physical activity, body image, nutritional status, adults, sex differences

## Abstract

The association of body image dissatisfaction and obesity with physical activity is likely to differ according to gender. To investigate this hypothesis, we conducted a cross-sectional study among the ELSA-Brasil cohort members aged 34–65 years (n = 13,286). The body image dissatisfaction was present even among normal weight individuals of both sexes and was associated with lesser chances of practicing moderate physical activity in women and intense physical activity in men. Men and women with central obesity were less prone to practice physical activity of high or moderate intensity. Overweight and obese men were more likely to report vigorous physical activity while obese women were less likely to report this level of physical activity. Body images as well as nutritional status are related to physical activity in both sexes, but the association with physical activity differs by gender.

## 1. Introduction

Even though the importance of physical activity is widely recognized, millions of adults remain sedentary, resulting in alarming sedentary lifestyle rates [1]. Considering the world population, approximately 31% of the adults are physically inactive [2], but more than 60% of them are not sufficiently active in order to obtain health benefits, according to the World Health Organization [1,3]. In Brazil, estimates for all capitals and the Federal District, also show that only one third of adults practice the recommended amount of physical activity during their leisure time, being this percentage higher among men (41.2%) than among women (27.4%) [4].

Obesity and abdominal obesity are associated independently with morbidity and mortality [5] and physical activity is a key point to manage this worldwide epidemic [6]. The association between obesity and sedentary lifestyle is dynamic, for obesity can be either the consequence or the cause of sedentary behavior [7]. Physical inactivity contributes to weight gain, because it leads to an adverse energetic balance [8]. On the other hand, obese people tend to be less active, in part due to mobility problems caused by obesity and/or to embarrassment related to a negative body image [9,10]. A longitudinal study with a middle-aged cohort suggested that higher body weight, body mass index and fat mass may predict future sedentary behavior, independent of baseline confounding factors. Therefore, the increase in the prevalence of obesity also contributes to the rising of physical inactivity levels [7].

Body image can be defined as a multifaceted psychological construct that includes subjective attitudinal and perceptual experiences about one’s body, particularly its appearance [11]. The construction and perception of body image are strongly influenced by social standards and the media, which advertise almost unattainable perfect bodies in most Western countries [12]. Usually, women present more body weight/shape concerns than men [13] and tend to consider thinness as the preferred body image style [14]. Despite the concerns about body image are often associated with adolescence in the literature [11,14,15], these concerns also affect adults, especially women. One explanation for the women’s permanent concern with their body image would be the so-called “double standard of aging”. This concept suggests that modern urbanized societies allow two standards of male beauty (the boy and the man), but only one for females (the girl). Consequently, growing older for women in Western society means that they will have to cope with the stigma of unattractiveness simply because of the aging process [16]. 

Body image dissatisfaction occurs when the perceived body image and the ideal body image are not congruent [17]. Usually, the body image dissatisfaction is assessed by standardized questionnaires about body image perception [11,12] or by figure rating scale of silhouettes, from the thinnest to the largest body type [14,18]. Some investigators have already provided evidence that the body image dissatisfaction seems to influence the practice of physical activity in two senses. A positive body image may stimulate the engagement in and continued adherence to physical activity [19], as indicated in a cross-sectional analysis of 19,003 participants of the Aerobics Center Longitudinal Study (ACLS), in which the weight satisfaction was independently associated with being moderate to vigorously active [20]. 

In theory, individuals who feel dissatisfaction about their body image are more likely to engage in behaviors to fight the discomfort [18]. However, the literature has shown that a negative body image may decrease the motivation to practice physical activity [21]. Kruger *et al.* [22] observed in 10,021 participants of the National Physical Activity and Weight Loss Survey (NPAWLS) that white men and women who were somewhat or not satisfied with their body size were less likely to be regularly active.

Research on gender differences indicates that males and females exhibit different aspects that encourage the practice of physical activity. In general, men seek to gain muscular mass and do the activity for challenge or competition [23,24] and strength [23], which may explain the fact that they are more active and practice more vigorous activities [1]. Appearance, physical condition [24] and weight management [23] are the most common motives for women to do physical activity and they are more prone to choose and practice mild physical exercises, such as walking and dance to maintain or lose weight and gain firmness [25]. 

Few studies have investigated, simultaneously, whether body image dissatisfaction and obesity are associated with the practice of physical activity during leisure time and if these associations differ significantly among men and women. The majority of the published studies were carried out in high-income countries, where patterns of physical activity are different from those observed in developing countries, such as Brazil [3,22]. Most studies that also address body image refer to children and adolescents [11,14,15,26,27]. Moreover, body image might have even greater role in promoting physical activity in a tropical country, due to greater exposure and concern about body shape, as compared to temperate countries [25].

The present study investigates whether dissatisfaction with body image, obesity and central obesity are independently associated with physical activity and if such association is different among men and women participants in the Longitudinal Study of Adult Health (ELSA-Brasil). The main hypothesis is that moderate or intense physical activity is less frequent among individuals who are dissatisfied with their body image, and those who are obese and/or have greater central adiposity indicator. We believe that the dissatisfaction with own body image is more strongly related to the practice of physical activity among women than men, independent of nutritional indicators. Conversely, we think that anthropometric indicators are more frequently related to the practice of physical activity among men, independent of body image dissatisfaction. 

## 2. Methods 

### 2.1. Design

The present study is a cross-sectional study, which used the baseline participants of the multicenter ELSA-Brasil cohort. This cohort aims to investigate the incidence and progression of cardiovascular diseases and diabetes [28]. Public sector workers (15,105), active or retired, between the ages of 35 and 74 participated in the baseline of the study (2008–2010). Details on the cohort profile have been published elsewhere [29]. All subjects gave their informed consent for inclusion before they participated in the study. The study was conducted in accordance with the Declaration of Helsinki, and the protocol was approved by National Research Ethics Committee (CONEP N. 13065).

### 2.2. Participants

The present analysis included all individuals aged between 35 and 64 years who answered the questionnaire about physical activity and about the other variables of interest. The respondent mean age was 50 years (SD  =  0.6). The individuals with 65 years or more were excluded from this study (n = 1591) because the International Physical Activity Questionnaire (IPAQ) used to ascertain physical activity is not recommended for individuals aged 65 years and more [30]. We also excluded from the present analysis all individuals with a body mass index inferior to 17 kg/m^2^ (n = 21) due to the greater probability of health problems that can influence the physical activity practice among this group [31], as the presence of eating disorders [32,33], body image distortions [33] and weight loss attempts related to psychological symptoms [34]. 

### 2.3. Outcome of Interest

Trained personnel applied the Brazilian version of the short form IPAQ in face-to-face interviews in order to assess physical activity. The short form IPAQ was validated in several countries, including Brazil [35]. The procedures described in the *Guidelines for Data Processing and Analysis of the International Physical Activity Questionnaire* were employed in order to calculate the scores of physical activity, where each type of activity has a specific MET score: 3.3 METs for walking, 4.0 METs for moderate intensity activities and 8.0 METs for high intensity activities. Considering the quantity of days per week and minutes spent on each type of activity, we obtain the total of MET-min per week. 

The physical activity was categorized into: (a) low (those who do not exercise and who did not meet the criteria to be included into the other categories); (b) moderate (three or more days of high intensity activity during at least 20 min each day; or five or more days of moderate intensity activity and/or walking during 30 min per day; or five or more days of any combination between walking, moderate or intensity activity, reaching a minimum of 600 MET-min/week); and (c) high (high intensity activity during three days a week, reaching a minimum of 1500 MET-min/week; or seven days of any combination between walking and moderate or high intensity activity, reaching a minimum of total physical activity of 3000 MET-min/week). 

### 2.4. Explanatory Variables of Interest 

The body image was assessed according to the following questions: “*Which figure better represents your body today?*” (current body image); and “*Which figure better represents the body you would like to have?*” (ideal body image). In order to answer such questions, the participant was oriented to choose one of the 15 figures presented in a scale of silhouettes numbered in increasing order—from the slimmest (number 1) to the largest (number 15) silhouette [36]. The scale of silhouettes used in this study was developed by Kakeshita [36] following Gardner’s recommendations [37,38] and has demonstrated validity and reliability for use in the Brazilian population [39,40]. Although the original scale had 9 figures for each sex, the ELSA-Brasil version was expanded to 15 figures as the validation study indicated that subjects identified themselves with figures outside the limits of the original scale.

Body image dissatisfaction was obtained by subtracting the number corresponding to the ideal body image from the number corresponding to the current body image. When the difference was equal to zero, the individual was classified as *satisfied* and, if smaller than zero, *dissatisfied due to slimness*.When the result was higher than zero, the participants were classified as *dissatisfied due to overweight* [9].

The nutritional status of the participants is represented by the body mass index (BMI), calculated as kg/m^2^, and the central obesity. The weight was measured by a platform scale (Toledo^®^, São Bernardo do Campo, São Paulo, Brazil) with 50 g precision. The height was measured by a stadiometer (Seca^®^, Hamburg, Germany) with 0.1 cm precision. The BMI was categorized into: normal weight (<25 kg/m^2^); overweight (25.0 to 29.9 kg/m^2^); and obesity (>30 kg/m^2^).

The central obesity was evaluated by the waist to height ratio, which was calculated using waist circumference (cm) and the height (m). The circumference of the waist was measured by a non-extensible anthropometric tape (Mabis^®^, Waukegan, IL, USA) with 0.1 cm precision. The waist/height ratio (WHR) was categorized into: less than 0.5 (absence of central obesity); higher or equal to 0.5 (presence of central obesity) [41].

### 2.5. Adjustment Variables

The following variables were considered as potential confounding factors in the sex-specific analysis: age (categorized into 35 to 44 years, 45 to 54 years and 55 to 64 years in the univariate analysis and continuous, in the multivariate analysis), *per capita* family income, divided into quintiles (1st quintile: under U.S. $259.53; 5th quintile: over U.S. $980.77), schooling in years of school (<11 years; 11 to 14 years; >15 years), smoking (never smoked; former smoker; smoker) and mental disorder (no; yes).

The presence of mental disorder was verified by means of the *Clinical Interview Schedule Revised* (CIS-R), which evaluates the presence of 14 different symptoms during the week prior to the interview. The complete version of the CIS-R includes 14 sections which cover non-specific symptoms. The presence of 12 or more symptoms characterized the presence of common mental health disorder [42]. 

The presence of common mental health disorders was used for adjustment due to its association with physical activity [43] and body image perception [44]. Smoking was considered due to its association with body weight [45] and behaviors related to health, such as physical activity [46]. 

### 2.6. Statistical Analysis

After the descriptive analysis, the associations between the co-variables (explanatory and adjustment variables) and the outcome variable were estimated by the chi-square test, with a 5% significance level. The magnitude of the crude and adjusted associations between the physical activity level and the co-variables was estimated by the *odds ratio* and its 95% confidence interval, obtained by multinomial logistic regression. The reference category was low intensity physical activity and all the analyses were stratified by sex. After the univariate analysis, the multivariate analysis was carried out in order to test the independent associations between BMI, central obesity and body image dissatisfaction (explanatory variables) and the physical activity level. In order to include the variable in the multivariate analysis, we considered a *p*-value < 0.20 in the univariate analysis [47]. In all the other analyses, a 5% significance level was used as criterion to reject the null hypothesis. The analyses were carried out in the program Stata 12.0 (Stata Corporation, College Station, TX, USA).

## 3. Results 

Among the 13,286 participants studied, most were women (54.4%), aged between 45 and 54 (44%) and had more than 15 years of schooling (52.2%). With regard to physical activity level, 77.6% of the individuals practiced low intensity physical activity. The prevalence of individuals who practiced high intensity physical activity was greater among men than among women (Table 1).

The great majority of the participants (85.9%) was dissatisfied with their body image, 74.2% presented central obesity, 39.9% were overweight and 22.9% were obese. The prevalence of dissatisfaction due to slimness was higher among men and the proportion of dissatisfaction due to overweight was higher among women (Table 1), as well as the prevalence of normal weight, obesity and the presence of central obesity. 

**Figure 1:**
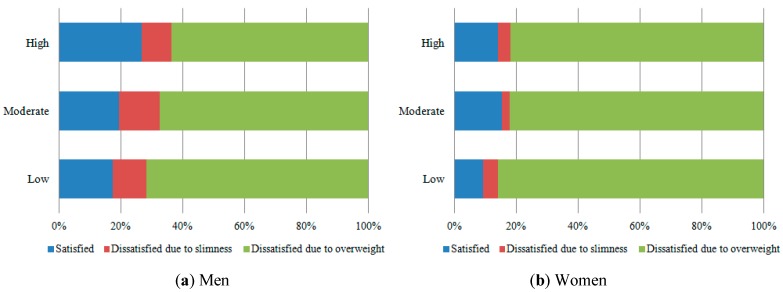
Prevalence of body dissatisfaction among men and women, according to the physical activity (35–64 years-old). ELSA-Brasil (2008–2010).

**Figure 2:**
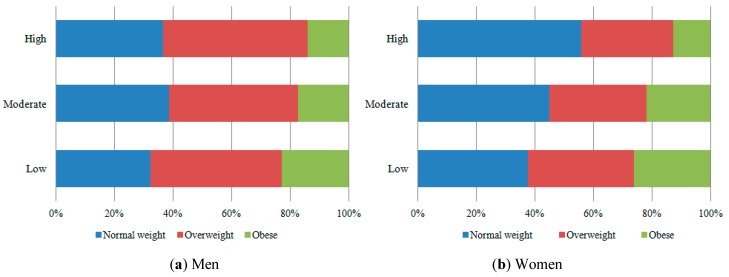
Prevalence of BMI among men and women, according to the physical activity (35–64 years-old). ELSA-Brasil (2008–2010).

The frequency of men satisfied with their body image was greater as the intensity of physical activity increased. The women who practiced low level of physical activity had the greatest frequency of obesity (Figure 2) and dissatisfaction due to overweight (Figure 1). 

In the univariate analysis, for both men and women, the odds of practicing moderate physical activity was higher among those who were aged between 55 and 64 years (when compared to younger age ranges); decreased with the reduction of the per capita household income and schooling level and was lower among those who presented overweight and obesity, central obesity, common mental disorders and smokers. With regard to body image dissatisfaction, among men, the chances of practicing high intensity physical activity were lower for those who presented body image dissatisfaction; among women, these chances were lower for those dissatisfied, regardless the physical activity level (Table 2). 

The pattern of the associations for those who practiced high intensity physical activity was similar to the one described for moderate intensity activity, except for age and body image dissatisfaction. Among men, the chances of practicing high intensity activity decreased with age. In both sexes, the odds of engaging in high intensity activity was smaller among those dissatisfied compared to the satisfied ones (Table 2).

In the multivariate analysis, practicing moderate physical activity remained associated with body image dissatisfaction among women. In comparison with those satisfied with their body image, the chances of engaging in moderate activity were smaller among those dissatisfied due to slimness and overweight. Even though body dissatisfaction was not statistically associated with the practice of moderate physical activity, the chances of practicing moderate activity were smaller in obese men (Table 3). 

**Table 1:** Distribution of the general characteristics of the participants (35–64 years-old), according to sex. ELSA-Brasil (2008–2010).

Variables	n (%)	Men (%) (n = 5997)	Women (%) (n = 7289)
*Physical activity levels*			
Low	10310 (77.60)	43.28	56.72
Moderate	1677 (12.62)	48.90	51.10
High	1299 (9.78)	55.04	44.96
*Age (years)*			
35 to 44	3335 (24.71)	46.75	53.25
45 to 54	5930 (43.93)	45.18	54.82
55 to 64	4233 (31.36)	43.80	56.20
*Per capita household income*			
5th quintile	2686 (19.99)	42.52	57.48
4th quintile	2500 (18.60)	42.92	57.08
3th quintile	2633 (19.59)	44.51	55.49
2th quintile	2871 (21.36)	44.76	55.24
1th quintile	2749 (20.46)	50.71	49.29
*Schooling (years)*			
>15	11934 (88.41)	42.99	57.01
11 to 14	868 (6.43)	58.06	41.94
<11	696 (5.16)	65.66	34.34
*Body image dissatisfaction*			
Satisfied	1900 (14.08)	59.63	40.37
Dissatisfied due to slimness	986 (7.30)	67.65	32.35
Dissatisfied due to overweight	10612 (78.62)	40.44	59.56
*BMI* **^a^**			
Normal weight	5021 (37.20)	41.07	58.93
Overweight	5382 (39.87)	51.02	48.98
Obese	3095 (22.93)	41.49	58.51
*Central obesity* **^b^**			
Absent	3477 (25.76)	38.19	61.81
Present	10,021 (74.24)	47.54	52.46
*Smoking*			
Never smoked	7739 (57.33)	40.73	59.27
Former smoker	3920 (29.04)	51.99	48.01
Smoker	1839 (13.62)	49.05	50.95
*Common mental disorder*			
No	9752 (72.25)	50.53	49.47
Yes	3746 (27.75)	31.07	68.93

Notes: For all the variables, the *p* value resulting from the Pearson chi-square test was *p* = 0.000. **^a^** Body mass index; **^b^** waist to height ratio > 0.5.

The chances of practicing of high intensity activity were associated with body image dissatisfaction only among men: they were lower for those dissatisfied. No significant associations between practicing high intensity activity and body image dissatisfaction were observed among women. BMI presented statistically significant associations with high intensity physical activity in both sexes, although in opposite directions. Men who presented overweight had higher chances of practicing high intensity activity when compared to normal weight individuals. In contrast, obese women, when compared to normal weight ones, presented lower odds of engaging in high intensity physical activity (Table 3). The frequency of moderate or high intensity activity was associated with central obesity in both sexes and was lower among those who presented an increased WHR (Table 3). 

**Table 2:** Crude *Odds Ratios* for physical activity, according to sociodemographic, body and behavioral characteristics, in adults (35–64 years-old), categorized by sex. ELSA-Brasil (2008–2010).

Variables	Men			Women	
	Physical Activity Levels			Physical Activity Levels	
	Moderate OR (95% CI)	High OR (95% CI)		Moderate OR (95% CI)	High OR (95% CI)
*Age (years)*					
35 to 44	1.00	1.00		1.00	1.00
45 to 54	1.09 (0.90–1.34)	0.76 (0.63–0.91) **^a^**		1.18 (0.96–1.44)	0.86 (0.69 – 1.06)
55 to 64	1.46 (1.19–1.79) ^**a**^	0.52 (0.42–0.64) ^**a**^		1.73 (1.42–2.12) ^**a**^	0.89 (0.71 – 1.11)
*Per capita household income*					
5th quintile	1.00	1.00		1.00	1.00
4th quintile	0.97 (0.77–1.22)	0.65 (0.51–0.82) ^**a**^		0.83 (0.67–1.02)	0.58 (0.46–0.74) ^**a**^
3rd quintile	0.75 (0.60–0.95) ^**a**^	0.58 (0.46–0.74) ^**a**^		0.68 (0.55–0.84) ^**a**^	0.51 (0.40–0.64) ^**a**^
2nd quintile	0.55 (0.44–0.70) ^**a**^	0.36 (0.28–0.46) ^**a**^		0.53 (0.42–0.66) ^**a**^	0.30 (0.23–0.40) ^**a**^
1st quintile	0.43 (0.34–0.55) ^**a**^	0.30 (0.23–0.39) ^**a**^		0.36 (0.28–0.46) ^**a**^	0.15 (0.10–0.21) ^**a**^
*Schooling (years)*					
>15	1.00	1.00		1.00	1.00
11 to 14	0.54 (0.39–0.74) ^**a**^	0.46 (0.32–0.66) ^**a**^		0.62 (0.42–0.91) ^**a**^	0.29 (0.15–0.55) ^**a**^
<11	0.50 (0.36–0.70) ^**a**^	0.30 (0.20–0.47) ^**a**^		0.61 (0.38–0.97) ^**a**^	0.12 (0.04–0.55) ^**a**^
*Body image dissatisfaction*					
Satisfied	1.00	1.00		1.00	1.00
Dissatisfied due to slimness	1.09 (0.83–1.43)	0.57 (0.42–0.77) **^a^**		0.33 (0.21–0.54) **^a^**	0.58 (0.36–0.94) **^a^**
Dissatisfied due to overweight	0.84 (0.70–1.02)	0.57 (0.48–0.69) **^a^**		0.58 (0.47–0.71) **^a^**	0.63 (0.49–0.80) **^a^**
*BMI*					
Normal weight	1.00	1.00		1.00	1.00
Overweight	0.82 (0.70–0.97) **^a^**	0.97 (0.82–1.16)		0.75 (0.64–0.89) **^a^**	0.57 (0.47–0.69) **^a^**
Obesity	0.62 (0.50–0.77) **^a^**	0.53 (0.42–0.68) **^a^**		0.69 (0.57–0.84) **^a^**	0.33 (0.25–0.42) **^a^**
*Central obesity*					
Absent	1.00	1.00		1.00	1.00
Present	0.71 (0.60–0.85) ^**a**^	0.48 (0.41–0.58) ^**a**^		0.71 (0.61–0.83) ^**a**^	0.41 (0.34–0.48) ^**a**^
*Smoking*					
Never smoked	1.00	1.00		1.00	1.00
Former smoker	1.01 (0.85–1.20)	0.88 (0.72–1.06)		1.19 (1.01–1.41)	1.23 (1.00–1.51)
Smoker	0.60 (0.47–0.78) ^**a**^	0.52 (0.39–0.70) ^**a**^		0.64 (0.49–0.83) ^**a**^	0.64 (0.46–0.89) ^**a**^
*Common mental disorder*					
No	1.00	1.00		1.00	1.00
Yes	0.72 (0.59–0.88) ^**a**^	0.59 (0.47–0.74) ^**a**^		0.60 (0.51–0.70) ^**a**^	0.45 (0.37–0.56) ^**a**^

Notes: **^a^** Significant associations (*p* < 0.05); BMI: body mass index.

**Table 3:** Adjusted **^b^** *Odds Ratios* for physical activity, according to body characteristics among adults (35–64 years-old), categorized by sex. ELSA-Brasil (2008–2010).

Variables	Men			Women	
	Physical Activity Levels			Physical Activity Levels	
	Moderate OR (95% CI)	High OR (95%CI)		Moderate OR (95% CI)	High OR (95% CI)
*Body image dissatisfaction*					
Satisfied	1.00	1.00		1.00	1.00
Dissatisfied due to slimness	1.13 (0.85–1.50)	0.57 (0.42–0.79) **^a^**		0.37 (0.22–0.59) **^a^**	0.68 (0.42–1.12)
Dissatisfied due to overweight	0.95 (0.75–1.19)	0.63 (0.50–0.79) **^a^**		0.69 (0.54–0.87) **^a^**	1.01 (0.77–1.34)
*BMI*					
Normal weight	1.00	1.00		1.00	1.00
Overweight	0.96 (0.77–1.21)	1.78 (1.38–2.30) **^a^**		0.89 (0.72–1.10)	0.84 (0.65–1.07)
Obese	0.75 (0.57–0.98) **^a^**	1.11 (0.80–1.54)		0.90 (0.71–1.15)	0.57 (0.41–0.79) **^a^**
*Central obesity*					
Absent	1.00	1.00		1.00	1.00
Present	0.75 (0.59–0.96) **^a^**	0.44 (0.34–0.58) **^a^**		0.79 (0.63–0.98) **^a^**	0.60 (0.46–0.76) **^a^**

Notes: **^a^** Significant associations (*p* < 0.05); **^b^** Adjusted by age (continuous), *per capita* household income, schooling, smoking and common mental disorder; BMI: body mass index.

## 4. Discussion 

The results presented reveal that the prevalence of high and moderate intensity physical activity are very low, especially the high intensity activity among women. Also, the results showed that the presence of obesity in women and central obesity in both gender was associated with lower chances of practicing exercise, corroborating with our original hypothesis. But, unlike expected, the presence of overweight in men was associated with greater chances of practicing high intensity physical activity. Moreover, our findings confirm the other original hypothesis: that body image dissatisfaction is associated with smaller chances of practicing physical activity both in women and in men. However, in contrast with our hypothesis, this association was stronger among men. Other authors [9,22] also described that greater discrepancies between current and ideal body image were related to lower levels of physical activity during leisure time among men and women. 

Our results show that the direction of the association between BMI and the practice of physical activity is not the same for men and women. While being overweight increased the chances practicing high intensity physical activity among men, obesity was associated with smaller chances of practicing it among women. Obesity was also associated with less chance of practicing moderate physical activity in men. Central obesity was the only measure associated with both physical activity level: was negatively correlated to moderate and high intensity activity among men and women. 

As found in our study, there are evidences that the practice of physical activity is more frequent among men [25,48] and that women are more physically inactive [49]. A study with adults, using the long version of the IPAQ, found higher levels of physical activity among men, both in terms of duration and intensity of the exercise [1]. Low intensity activities, such as walking, dance and gymnastics prevail among the female sex [49]. Although, women’s interest in high intensity activity, which result in a more athletic and muscular silhouette, seems to have recently increased in Brazil [25] and in other countries [50]. 

The prevalence of obesity and overweight observed in this study reflects the general tendencies in the country. In 2013, a countrywide telephone interview survey (VIGITEL) [4] also revealed that the prevalence of overweight was higher among men (54.7% *vs*. 47.4%); whereas the prevalence of obesity was exactly the same among both sexes (17.5%). Our findings corroborate other studies that show that women tend to express more dissatisfaction with their body than men, especially due to overweight [51,52]. Women are generally concerned with reaching the ideal of slimness imposed by media and society, stressing the dissatisfaction with overweight. This was the observed among a cohort of North American health professionals, where the women reported to see themselves more overweight than the men, even the ones with normal weight [51]. Men, on the other hand, seek better physical conditioning and a more muscular appearance [22,53], which may explain the higher prevalence of dissatisfaction due to slimness among men found in this study. 

The association between physical activity and BMI may vary. Overweight and obesity can either be the result of physical inactivity as may promote it, especially in advanced cases of obesity in which there are physical limitations and constraints related to the appearance. [9,10]. In the present study, obese women presented smaller chances of practicing high intensity physical activity, and this finding is corroborated by *The Transition and Health During Urbanisation of South Africans (THUSA)* study, which revealed a strong association between physical inactivity and obesity among women [54]. In the present study, it is equally likely that the small frequency of physical activity led to obesity among women, due to insufficient energy expenditure for the loss or maintenance of body weight, or that prior overweight demotivated the practice of physical activity. In a longitudinal study with 3042 adults, overweight predicted the increase of physical inactivity after a 5-year follow-up [3]. 

Differently from women, overweight was associated to greater chances of high intensity physical activity among men. In this group, specifically, overweight may indicate a higher proportion of lean body mass and not excess of body fat. The BMI presents a good correlation with body fat percentage [55], considering the population characteristics. However, this index does not distinguish adequately between lean and fat body mass and may be a less precise indicator of adiposity among men [56]. Thus, it is possible that part of the association between overweight and greater frequency of high intensity activity observed in our study among men is related to higher percentages of lean body mass. This conjecture is substantiated by the smaller frequency of high or moderate physical activity among men with central obesity. In conclusion, overweight among men who practice high intense activity may express greater muscularity in this group. 

The results show an association between body image dissatisfaction and lower chances of practicing moderate intensity physical activity among women. Moreover, as the obesity was correlated with lower chances of high intensity physical activity among women, we can infer that both objective (BMI and waist/height ratio) and subjective aspects (body image dissatisfaction) seem to be associated with the decrease of physical activity among women and, therefore, do not promote a healthy behavior. One of the main motives to exercise in women is the appearance [24], but we were not expecting that the body image dissatisfaction could be related to the increase of physical activity. Moreover, what is actually found in the literature are studies like the one of Anton *et al.* [9], that found an inverse dose-response gradient connecting the practice of physical activity and the level of discrepancy between current and ideal body image. 

Roy and Payette [16] conducted a systematic review in which they report that older people’ body image importance related to body competence increases whereas the one attributed to physical appearance decreases. Thus, it is possible that older participants of the present study are experiencing a transition in their body image judgment and this may lead to changes in what motivate them to do physical activity as the years go by. 

In contrast with our hypothesis, not only objective body indicators, but also body image dissatisfaction, were associated with high intensity physical activity among men. Some authors suggest that men tend to better accept their bodies, since they are less prone to social pressure and stigmas related to body weight [13,57]. Tiggemann *et al.* [58] defend that body weight and body perception are also aspects that determine men’s body image, and may influence masculine behavior concerning physical activity. Recently, men have revealed dissatisfaction with their body and sought a mesomorph body ideal: wide shoulders, well developed upper body, flat abdomen and narrow hips [58,59]. Nonetheless, what we observe does not suggest that body dissatisfaction promotes physical activity; on the contrary, our results indicate that the dissatisfaction is related to smaller chances of practicing physical activity. This means that body dissatisfaction, even due to slimness, does not seem to motivate the quest for the socially idealized masculine body. The mesomorph body model is often pursued by young adults [53], which suggests that the more advanced age range of the present cohort may have influenced the direction of the association found between body dissatisfaction and physical activity among men. Furthermore, the scale of silhouettes used in the present study does not indicate the muscularity of the body, since it was built based only on body mass index [36]. 

The univariate analysis confirmed the associations, already described by many authors, between decrease in physical activity and ageing [30], lower income and schooling level [2,60], as well as smoking [46] and common mental health disorder [43]. The adjustment for these variables showed that both objective indicators of body weight and subjective indicator of dissatisfaction with body image are not confounded by them, reinforcing our results. 

This study had limitations. The short version of the IPAQ evaluates only physical activity during leisure time, and does not measure physical activity in different domains, such as labor and transport. Although it is the most commonly used instrument in large scale surveys, another limitation of the short form of IPAQ is its use to estimate physical activity levels, since it overestimates physical activity when compared to other questionnaires [61] and to the long version of IPAQ [62]. 

In addition to the specificities regarding body weight distribution, the age range from 65 to 74 years was not included in the study because the silhouette scale instrument was not designed for this particular group, whose body morphology can be different. Furthermore, the reliability of the body silhouette scales used by ELSA-Brasil was lower among this age range when compared to the others [39]. Similarly, the IPAQ requires adaptations in its structure and application in order to be implemented with elder individuals [63]. The present study has a cross sectional design and, therefore, does not have the aim of testing the causal relationship between the factors of interest and physical activity. Notwithstanding, this design supplies evidences concerning potential variables which could be useful in order to plan interventions and to aid creating hypothesis for the longitudinal analysis. 

In summary, our results reveal that body perception as well as objective indicators are related to the practice of physical activity in both sexes, but that such associations are different among men and women, according to the intensity of the physical activity practiced by the participants of the cohort. It is important to further understand the factors that are associated with body dissatisfaction and verify whether they are motivators or inhibitors of the longitudinal increase of physical activity. The low prevalence of physical activity among men and women participating in the study, similar to the one found among the population in general, is worrying, since the participants of ELSA-Brasil present a schooling level far higher to that of the average Brazilian population. This reinforces the need to extend the knowledge about factors that promote and inhibit physical activity, which is so vital to physical and mental health. 

## 5. Conclusions

In summary, our results reveal that body perception as well as objective indicators are related to the practice of physical activity in both sexes, but that such associations are different among men and women, according to the intensity of the physical activity practiced by the participants of the cohort. 

A challenge in health promotion is to improve the participant’s perception of body image while promoting physical activity. It is important to further understand the factors that are associated with body dissatisfaction and verify whether they are motivators or inhibitors of the longitudinal increase of physical activity.

Also, with the advent of searching a muscular body by men, and also women, in tropical countries, it would be important to develop a figure rating scale of silhouettes that consider not only the BMI of individuals but also the distribution of muscles and fat in the body.

The low prevalence of physical activity among men and women participating in the study, similar to the one found among the population in general, is worrying, since the participants of ELSA-Brasil present a schooling level far higher to that of the general Brazilian population. Prevention programs should encourage these adults to be more physically active, highlighting and promoting a healthy lifestyle. As physical activity so vital to physical and mental health, this scenario reinforces the need to extend the knowledge about other possible factors that promote and inhibit physical activity in middle-age adults and whether these factors can be different across life span. 
